# The rumen liquid metatranscriptome of post-weaned dairy calves differed by pre-weaning ruminal administration of differentially-enriched, rumen-derived inocula

**DOI:** 10.1186/s42523-021-00142-z

**Published:** 2022-01-05

**Authors:** Tansol Park, Laura M. Cersosimo, Wendy Radloff, Geoffrey I. Zanton, Wenli Li

**Affiliations:** 1 USDA-Agricultural Research Service, Dairy Forage Research Center, Madison, WI USA; 2 Department of Animal Science and Technology, Chung-Ang University, Anseong, Gyeonggi-do Korea

**Keywords:** Adult rumen fluid inoculation, Metatranscriptomics, Protozoa, Dairy calves, Microbiome network

## Abstract

**Background:**

Targeted modification of the dairy calf ruminal microbiome has been attempted through rumen fluid inoculation to alter productive phenotypes later in life. However, sustainable effects of the early life interventions have not been well studied, particularly on the metabolically active rumen microbiota and its functions. This study investigated the sustained effects of adult-derived rumen fluid inoculations in pre-weaning dairy calves on the active ruminal microbiome of post-weaned dairy calves analyzed via RNA-sequencing.

**Results:**

Two different adult-derived microbial inocula (bacterial- or protozoal-enriched rumen fluid; BE or PE, respectively) were administered in pre-weaned calves (3–6 weeks) followed by analyzing active rumen microbiome of post-weaned calves (9 weeks). The shared bacterial community at the genus level of 16S amplicon-seq and RNA-seq datasets was significantly different (*P* = 0.024), 21 out of 31 shared major bacterial genera differed in their relative abundance between the two analytic pipelines. No significant differences were found in any of the prokaryotic alpha- and beta-diversity measurements (*P* > 0.05), except the archaeota that differed for BE based on the Bray–Curtis dissimilarity matrix (*P* = 0.009). Even though the relative abundances of potentially transferred microbial and functional features from the inocula were minor, differentially abundant prokaryotic genera significantly correlated to various fermentation and animal measurements including butyrate proportion, body weight, and papillae length and counts. The overall microbial functions were affected quantitatively by BE and qualitatively by PE (*P* < 0.05), and this might be supported by the individual KEGG module and CAZymes profile differences. Exclusive networks between major active microbial (bacterial and archaeal genera) and functional features (KEGG modules) were determined which were differed by microbial inoculations.

**Conclusions:**

This study demonstrated that actively transcribed microbial and functional features showed reliable connections with different fermentations and animal development responses through adult rumen fluid inoculations compared to our previous 16S amplicon sequencing results. Exclusive microbial and functional networks of the active rumen microbiome of dairy calves created by BE and PE might also be responsible for the different ruminal and animal characteristics. Further understanding of the other parts of the gastrointestinal tract (e.g., abomasum, omasum, and small intestine) using metatranscriptomics will be necessary to elucidate undetermined biological factors affected by microbial inoculations.

**Supplementary Information:**

The online version contains supplementary material available at 10.1186/s42523-021-00142-z.

## Background

After raising questions about the difficulties of manipulating the stable and resilient adult rumen microbiota of dairy cattle [1, 2], rumen microbiologists have turned attention to targeting the simple and adjustable young dairy calves’ microbiota to enhance animal productivity. Starting from the non-functional and sterile gut [3], dairy calves acquire a variety of anaerobic microbiota, including fibrolytic bacteria, particularly following the drastic dietary shift while adapting to solid feeding [4, 5]. The establishment of adequate multi-kingdom microbiota for dairy calves allows them to use recalcitrant fibers and to maintain the health of the rumen ecosystem [6, 7].

Adult-derived rumen microbial inoculation of dairy calves has been studied as a potential probiotic application facilitating the transfer of numerous microbes at once. While adult-rumen microbial inoculation studies with young ruminants didn’t significantly impact rumen microbial populations and animal growth measurements [8, 9], potential beneficial effects on gut health, fermentations, and rumen development have been demonstrated [8, 10].

Previous studies have used DNA amplicon sequencing approaches to describe microbial communities from the rumen contents or rumen epithelium of young ruminants [11–13]. In our previous studies, we investigated the effect of adult-derived rumen fluid inoculation with two different microbial inocula (bacterial- or protozoal-enriched rumen fluid; BE or PE, respectively) in pre-weaning calves (3–6 weeks) [14] followed by confirmation of the sustained effects of the inoculations in post-weaned calves (9 weeks of age) [15]. Though these studies have shown significant insights into the overall bacterial community structure of the rumen and digestive tract, DNA-based sequencing methods are not capable of measuring the active microbial communities, as they cannot distinguish between inactive, alive, dead, or lysed cells [16, 17]. In contrast, RNA-based methods enable the study of the structure and potential function of the active microbial community by capturing actively transcribed RNA species. Due to this advantage, mRNA, or total RNA-based sequencing library preparation and cDNA-based, 16S rRNA gene amplicon sequencing (16S amplicon-seq) techniques have been utilized to better understand the active rumen microbial community [17–20].

Few studies have described the transcriptome of rumen fluid from dairy cows. To the author’s knowledge, no studies have described the rumen fluid transcriptome of dairy calves dosed with bacterial- and protozoal-enriched rumen inocula. The objectives of our experiment were to use RNA-sequencing to determine if the BE and PE microbial inoculations during the pre-weaning period (3–6 weeks of age; [14]) affect the active bacterial and archaeal community structures, associated functional compositions, and their co-occurrence patterns in post-weaned dairy calves. As a follow-up to our previously published work, we also compare the results from RNA-seq and 16S amplicon-seq based analyses to assess the commonality and uniqueness of both methods.

## Methods

### Experimental design and calf management

The animal procedures, dietary conditions, inoculum preparations, and experimental designs were described in our previous study [15]. Briefly, calves were kept ciliate-free before the experiment by separating them from their dam at birth, received colostrum within 4 h after birth, and were housed in individual calf hutches at the US Dairy Forage Research Farm in Prairie du Sac, WI. Twenty Holstein bull calves were randomly assigned to one of four different types of ruminal inocula (1. autoclaved, clarified rumen fluid, 2. BE, 3. PE, 4. BE and PE). A detailed description of the collection, processing and separation of ruminal inoculum was detailed in our 2 previous papers [14, 15]. Briefly, rumen contents from 4, first lactation, ruminally cannulated cows were removed and either blended, strained, centrifuged, and combined to create the bacteria-enriched supernatant or strained, placed in a separatory funnel for 1 h, and the protozoa-enriched pellet was removed and combined to create the protozoa-enriched inoculum. Calves were orally dosed the respective treatment inocula once per week from 3 to 6 weeks of age.

### Sample collection

All the procedures to analyze animal measurements, papillae length and numbers, and rumen fermentation characteristics were measured in our previous study at 9 weeks of age [15]. Additionally, calf health (rectal temperatures, nasal discharge, eye, ear, and feces) were assessed at 9 weeks of age using the University of Wisconsin School of Veterinary Medicine’s Scoring Chart (https://fyi.extension.wisc.edu/heifermgmt/files/2015/02/calf_health_scoring_chart.pdf, accessed January 19, 2021) as previously described [14].

### RNA extraction and sequencing

Rumen fluid samples (3 mL) for RNA analyses were immediately frozen with liquid nitrogen and stored at − 80 °C. Total RNA was extracted from 200 μl frozen rumen fluid (from both the inoculum and rumen samples) using the AllPrep Power Viral Kit (Qiagen, Hildlen, Germany) with the β-mercaptoethanol and solution PV1 premix option. The RNA integrity number (RIN) was measured with an Agilent 2100 Bioanalyzer. The samples with RIN greater than 7 were pursued for further analysis. RNA sample concentration was checked using a Qbit 3.0 (Thermo Fisher, US). An amount of 500 ng of total RNA from each sample was used for RNA sequencing library preparation using the Illumina TruSeq mRNA kit (Cat. 20020594) and it’s protocol (TruSeq stranded mRNA reference guide 1000000040498, Illumina, US). Initial steps of mRNA enrichment (as described under the section of “Purify mRNA”, using Poly-T oligo attached magnetic beads) were skipped to maximize the capture of microbial RNA reads. The work flow started by combining 5 μl total RNA with the 13 μl of Fragment, Prime, Finish mix and continued with the Elution 2-Frag-Prime program on the thermal cycler as described in the protocol and the steps afterwards. The fragment size and distribution of prepared libraries were assessed using a Bioanalyzer DNA 1000 kit (Agilent Technologies, CA, US). Library pooling was done by the library concentration determined by a Kapa quant kit (Cat. KK4873). After initial pooling, a MiSeq run was done using a MiSeq nano kit (Cat. MS-103–1001). Further normalization of the library pooling was done using the index ratio obtained for each library calculated by the MiSeq run. After this step, the final, pooled libraries were sequenced on a NextSeq sequencer using high-output 150-cycle kits (Cat. 20024907) to generate 2 × 75 bp paired-end reads.

### Metatranscriptome analysis

Additional file 1: Fig. S1 provided the major analytic workflow for metatranscriptomics used in this study. The quality of RNA-seq raw reads was first checked using FastQC (https://www.bioinformatics.babraham.ac.uk/projects/fastqc/, accessed on 01/25/2019). To filter out the reads from the host, raw RNA-seq were first mapped to the host reference genome UMD 3.1 of *Bos taurus* (http://ccb.jhu.edu/software/tophat/igenomes.shtml, April 1st, 2019) using the STAR aligner [21]. Unmapped reads were considered to be of rumen microbial origin and used for the downstream microbial community analysis. The host genome-filtered reads were used for bacterial and archaeal taxonomic classification using Kraken2 (v.2.0.8-beta) [22] with a confidence score of 0.2. Kraken2 standard database was downloaded and built on 4th June 2021. Among the host genome-filtered reads, non-rRNA reads were separated from rRNA reads by SortMeRNA (version, 2.1b) [23] based on the reference rRNA databases provided by Silva (release 138) [24]. Sequences classified as chloroplast and mitochondria were excluded before downstream analysis. Raw-read counts at each taxonomic level (i.e., phylum and genus) were normalized per sample with the per million factor as described in [25]. Major classified taxa, defined as occupying over 0.01% average relative abundance in at least one of the treatments, were discussed in this study.

Alpha-diversity measurements including richness indices (number of observed genera and Chao1 richness estimates), Evenness, Shannon’s index, and Simpson’s index were analyzed using the rarefied read count table of classified bacterial and archaeal genera. The overall bacterial and archaeal communities shaped by microbial inoculations were visualized using principal coordinates analysis based on the Bray–Curtis and Jaccard distance matrices at the genus level.

FastSpar [26], which uses the SparCC algorithm to calculate correlation estimations for compositional data [27], analyzed co-occurrence and mutual exclusion networks among the major microbial and functional features (> 0.01% relative abundance in at least one of the inoculation conditions) represented by bacterial and archaeal genera and Kyoto Encyclopedia of Genes and Genomes (KEGG) modules, respectively. By comparing the significant interactions between the microbial inoculation treatments and their corresponded controls, exclusive features and interactions were selected using the R package, Co-expression Differential Network Analysis (CoDiNA) [28] and were visualized using Gephi [29].

Venn diagrams were used to visualize the number of shared and exclusively detected bacterial and archaeal genera, and KEGG modules between two types of microbial inoculums and their corresponding rumen liquid samples.

### Taxonomic analysis of 16S amplicon-seq dataset

To compare the taxonomic classification outputs between DNA-based and RNA-based datasets, previously published 16S amplicon-sequences [15] were re-classified against the Kraken reference dataset. First, amplicon sequence variants generated by DADA2 [30] from the previous study were classified using Kraken2 and the classification was subsequently transformed to QIIME2-readible taxonomy file. The newly curated taxonomy file was used to re-calculate the abundance of each taxon in each sample. Only the major classified phyla and genera (defined as occupying over 0.01% average relative abundance in at least one of the treatments), which were jointly found in both the 16S amplicon-seq and RNA-seq datasets, were further analyzed for both the individual taxa and overall bacterial communities at genus level between those datasets.

### Functional annotation based on the non-rRNAs

Non-rRNA reads, which were the remainders after filtering out the sequences mapped to the bovine genome and rRNA genes, were considered as microbial protein coding gene reads and were used to analyze the potential microbial functions. Following the analytic pipeline suggested in Dai et al. [31], non-rRNAs were searched against NCBI nonredundant protein sequences (downloaded and built on 20th May 2020) using blastx module in DIAMOND [32] with an E-value of 1e-5 and a bit score of 50 as a cutoff. The deduced protein sequences were extracted from best-hit and used to annotate KEGG ortholog profiles via the KEGG Automatic Annotation Server (KAAS) [33] using GHOSTX program to search prokaryotic representative gene sets and subsequently mapped to KEGG modules using the python script implemented in picrust2 package [34]. Carbohydrate-active enzymes (CAZymes) annotations were further searched using non-rRNAs of inoculum and rumen samples by dbCAN2 [35]. The CAZymes were reported when at least two out of three annotation tools [i.e., HMMER (http://www.hmmer.org), DIAMOND, and Hotpep [36]] detected the same annotations satisfying recommended search cutoffs. The relative abundance of each CAZyme was calculated based on the total number of annotated CAZymes.

### Statistical analysis

Inoculum treatments were denoted as follows; with or without BE inoculation: BE(+) or BE(−), respectively, and with or without PE inoculation: PE(+) or PE(−), respectively. Comparing bacterial and archaeal alpha-diversity measurements and the abundance of CAZymes between two types of the inoculums was done with PROC GLIMMIX procedure in SAS 9.3 (SAS Institute Inc., Cary, NC, USA) with the fixed effect of inoculum type and random effect of calf. Negative bionomial distribution was applied to the features with non-normal distribution. Effects of microbial inoculations (BE or PE) on the alpha-diversity measurements at genus level were statistically analyzed using the GLIMMIX procedure of SAS 9.3 and the model included fixed effects of BE and PE, and the interaction between BE and PE. LEfSe [linear discriminant analysis (LDA) effect size] [37] was used to examine the difference of normalized bacterial and archaeal abundances at both phylum and genus levels and normalized counts of KEGG modules and annotated CAZymes by microbial inoculations with an LDA score 2 as cutoff. Calf health assessments were also analyzed using LEfSe except rectal temperature. Permutational multivariate analysis of variance (PERMANOVA) analysis (vegan::Adonis with 9,999 random permutations) was computed to analyze the beta-diversity based on the Bray–Curtis and Jaccard distance matrices of bacteriota, archaeota and functional profiles of KEGG modules. The correlations of normalized abundance of major active bacterial and archaeal genera with the animal performance measurements, fermentation characteristics, and protozoal counts which have been previously reported in Park et al. [15] were determined based on the Pearson correlation coefficients (correlation coefficient, |*r*|≥ 0.6, *P* ≤ 0.05) using PROC CORR procedure in SAS 9.3 and subsequently visualized using the corrplot package in R (v3.5.0). Within each CoDiNA selected exclusive network, keystone microbial and functional nodes were chosen based on two of the centrality measurements (i.e., authority and eigenvector centrality) calculated using the built-in plugins implemented in Gephi [29]. Significance was declared at *P* ≤ 0.05 and trends at 0.05 < *P* ≤ 0.1.

## Results

### Calf health assessments

All the assessments including rectal temperatures, nasal discharges, eye, ear, respiratory and fecal scores of dairy calves were not significantly differed by BE and PE inoculations (*P* > 0.05) (Additional file 2: Table S1).

### Comparative analysis of rumen bacteria using RNA-seq and 16S amplicon-seq

**Fig. 1:**
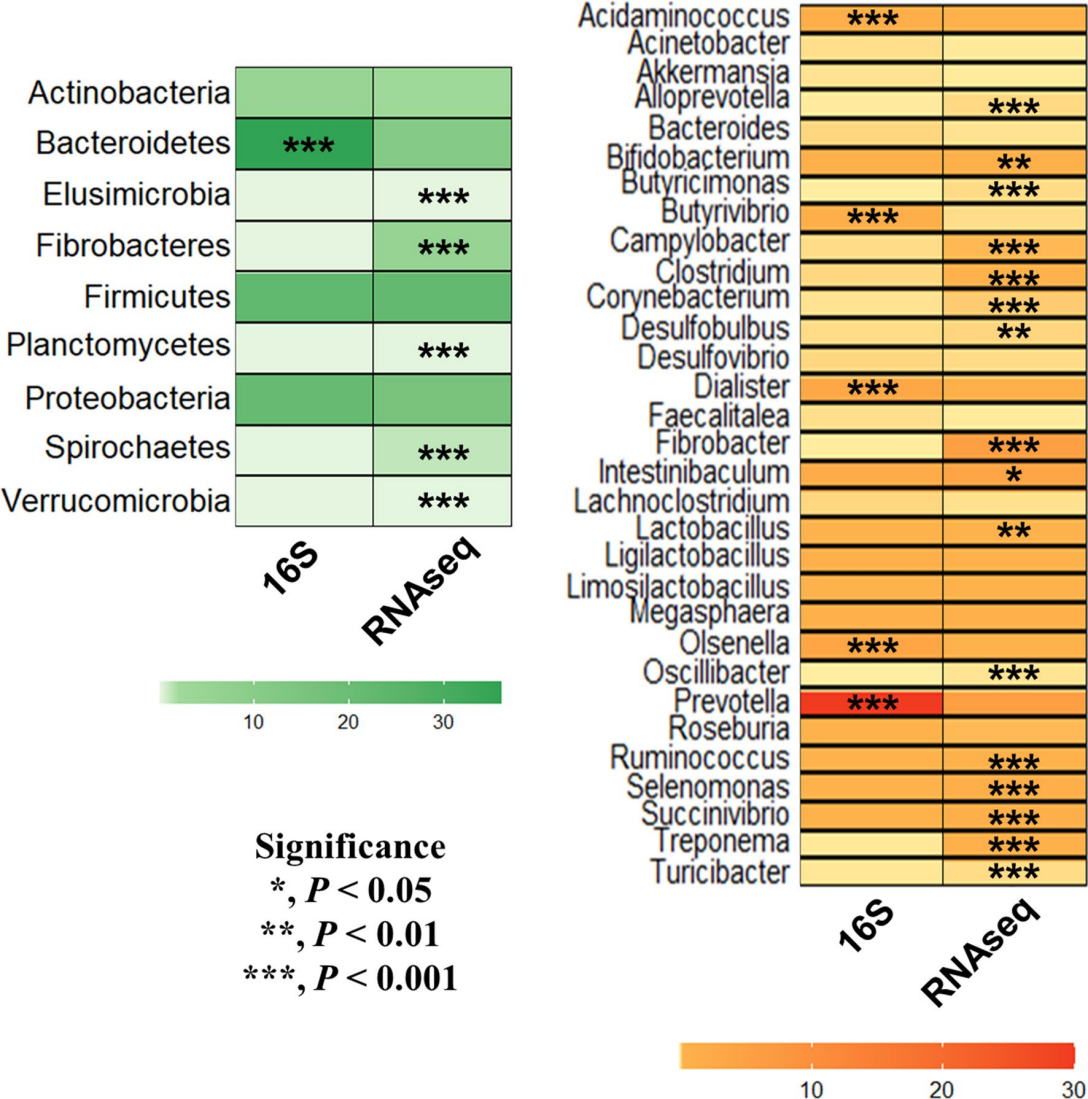
Differentially abundant major bacterial taxa at phylum and genus levels detected by both the 16S amplicon-seq and RNA-seq. Only the shared taxa between two different sequencing strategies and major taxa (occupying over 0.01% average relative abundance in at least one of the analytic pipelines) were selected for the comparison. The intensity of the color represents the relative abundance (%) based on the color key on the bottom

In total, there were 37 and 13 bacterial phyla and 1,207 and 106 bacterial genera classified from the RNA-seq and 16S amplicon-seq, respectively. Among the nine major bacterial phyla (defined as occupying over 0.01% average relative abundance in at least one of the analytic pipelines) detected by both of the RNA-seq and 16S amplicon-seq analytic pipelines in the rumen fluid samples of dairy calves, the relative abundance of Bacteroidetes was greater in 16S amplicon-seq dataset whereas the relative abundance of six phyla (Elusimicrobia, Fibrobacteres, Firmicutes, Planctomycetes, Spirochaetes, and Verrucomicrobia) were greater in RNA-seq dataset (Fig. 1). At genus level, 31 shared major bacterial genera represented 21.2 and 43.6% of the total bacteriota which were analyzed by RNA-seq and 16S amplicon-seq, respectively. Meanwhile, 1150 genera representing about 2.34% of overall bacteriota were uniquely identified in RNA-seq and 22 of those (*Lancefieldella*, *Collinsella*, *Berryella*, *Duncaniella*, *Sodaliphilus*, *Rufibacter*, *Cruoricaptor*, *Solitalea*, *Elusimicrobium*, *Bacillus*, *Staphylococcus*, *Eubacterium*, *Anaerostipes*, *Blautia*, *Coprococcus*, *Bulleidia*, *Planctopirus*, *Solimonas*, *Moraxella*, *Vibrio*, *Sphaerochaeta*, and *Ereboglobus*) were considered as major genera in active bacteriota, whereas no classified genera were uniquely identified in 16S amplicon-seq (data not shown). Except 10 genera (*Acinetobacter*, *Akkermansia*, *Bacteroides*, *Desulfovibrio*, *Faecalitalea*, *Lachnoclostridium*, *Ligilactobacillus*, *Limosilactobacillus*, *Megasphaera* and *Roseburia*), 21 out of 31 genera differed in their relative abundances between two analytic pipelines. Based on the Bray–Curtis dissimilarity matrices, the shared bacterial community at the genus level of 16S amplicon-seq and RNA-seq datasets was significantly different (*P* = 0.024) (Additional file 3: Fig. S2) and the overall bacterial community (based on Bray–Curtis distance matrix) between the two analytic pipelines were also significantly different (*P* = 0.027) (data not shown).

### The impact of adult rumen inoculation on the rumen bacterial diversity, archaeal diversity, and overall functional distribution

From the extracted RNA samples, with the average RNA integrity number of 7.4 ± 0.25 [(mean ± standard error of the mean (SEM)], an average of 59,153,238 ± 1,314,352 high-quality reads were generated with a range of 48,091,338 to 70,643,716 reads (Additional file 2: Table S2). For the inoculum samples, an average of 51,733,503 ± 5,150,354 high-quality reads were generated with a range of 42,515,561 to 61,110,565 reads (Additional file 2: Table S3). Following the quality filtering and removal of reads from host genome, 58,890,367 ± 1,309,652 host genome-filtered reads per sample were successfully classified. These classified reads comprised at least over 68.6% of the host genome-filtered reads per sample by Kraken2. Alpha- and beta-diversity analyses were done with rarefied genus level BIOM tables containing 5,333,080 and 584,744 counts for bacteriota and archaeota, respectively. Good’s coverage of genus level classification was higher than 99.9% in all RNA-seq samples.

**Fig. 2:**
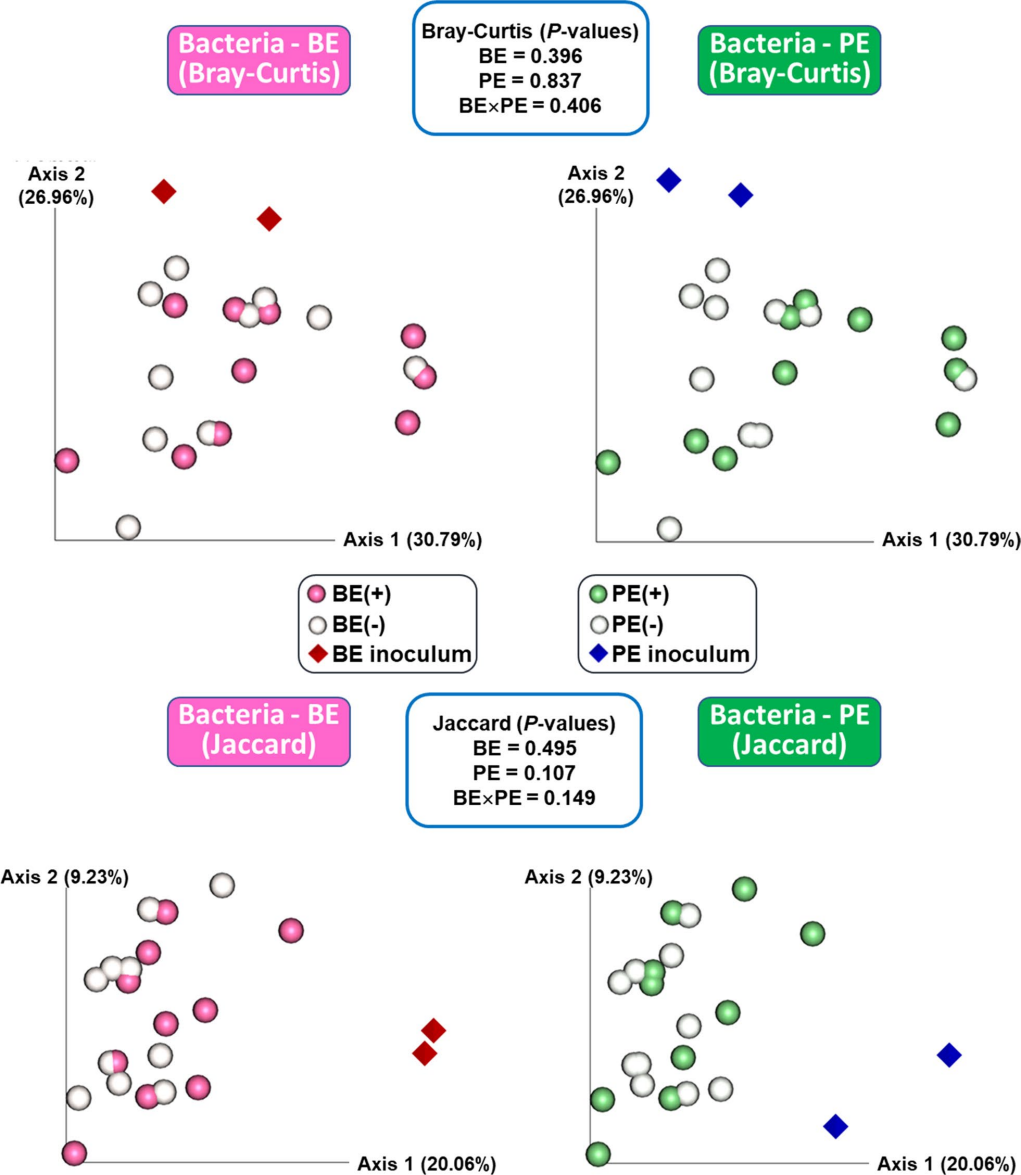
Principal coordinates analysis (PCoA) plot based on Bray–Curtis and Jaccard distance matrices representing overall rumen bacterial microbiota at the genus level in the liquid fraction of dairy calves differed by microbial inoculations with two types of inocula (BE and PE). BE, bacterial-enriched rumen fluid; PE, protozoal-enriched rumen fluid

Two inoculums had no differences for bacterial alpha-diversity measurements, whereas archaeal evenness and Shannon’s index was significantly higher in bacteria-enriched inoculum than those of the protozoa-enriched inoculum (*P* > 0.05) (Additional file 2: Table S4). No differences were observed for all microbial alpha-diversity measurements by both the microbial inoculations (Table 1). Based on the Bray–Curtis (quantitative) and Jaccard (qualitative) distance matrices, overall bacterial microbiota did not differ by BE and PE inoculations (*P* > 0.1; Fig. 2), whereas archaeal microbiota was significantly different between that of BE(+) and BE(−) calves based on the quantitative matrix (*P* = 0.009; Additional file 4: Fig. S3).

**Table 1:** Alpha diversity measurements in microbial inoculation treatments

Diversity measurements	Bacterial-enriched (BE)		Protozoal-enriched (PE)		SEM	*P*-values		
	+	−	+	−		BE	PE	BE × PE
**Bacterial alpha-diversity**								
Observed genera	525	507	533	499	17.465	0.643	0.367	0.906
Chao1 estimates	668	663	685	646	22.106	0.923	0.416	0.762
Evenness	0.328	0.360	0.331	0.356	0.011	0.155	0.265	0.660
Shannon's index	2.956	3.228	2.994	3.190	0.101	0.198	0.349	0.641
Simpson's index	0.756	0.810	0.762	0.804	0.019	0.161	0.279	0.690
**Archaeal alpha-diversity**								
Observed genera	11	10	11	9	0.505	0.295	0.219	0.923
Chao1 estimates	12	10	12	10	0.827	0.306	0.306	0.808
Evenness	0.362	0.309	0.334	0.337	0.025	0.326	0.956	0.910
Shannon's index	1.224	0.995	1.137	1.082	0.088	0.225	0.766	0.987
Simpson's index	0.438	0.349	0.393	0.393	0.036	0.258	0.997	0.973

At the phylum level, Proteobacteria was dominant in PE(−) calves [PE(+): 12.980 ± 1.470% and PE(−): 18.474 ± 2.327%, *P* = 0.049; Table 2]. *Bifidobacterium* and two genera within Firmicutes (*Anaerostipes* and *Oscillibacter*) were differentially abundant in BE(−) calves. For PE, the relative abundance of *Eubacterium* and *Desulfobulbus* was greater in PE(+) calves, whereas *Roseburia* and *Megasphaera* within Firmicutes and *Desulfovibrio* and *Moraxella* within Proteobacteria were dominant in PE(−) calves.

**Table 2:** Differentially abundant bacterial taxa (phyla and genera) by microbial inoculations

Dominance	Phylum	Genus	Relative abundance (%)		SEM	*P*-value
			BE(+)	BE(−)		
**By bacterial-enriched rumen fluid inoculation**						
BE(−)	Actinobacteria	*Bifidobacterium*	0.550	1.015	0.157	0.041
	Firmicutes	*Anaerostipes*	0.001	0.018	0.006	0.005
		*Oscillibacter*	0.008	0.012	0.002	0.049

Dominance	Phylum	Phylum	Relative abundance (%)		SEM	*P*-value
			PE(+)	PE(−)		
**By protozoal-enriched rumen fluid inoculation**						
PE(−)	Proteobacteria	-	12.980	18.474	1.480	0.049

Dominance	Phylum	Genus	Relative abundance (%)		SEM	*P*-value
			PE(+)	PE(−)		
PE(+)	Firmicutes	*Eubacterium*	0.083	0.026	0.014	0.049
	Proteobacteria	*Desulfobulbus*	0.037	0.012	0.005	0.019
PE(−)	Firmicutes	*Roseburia*	0.014	0.094	0.017	0.019
		*Megasphaera*	0.379	0.790	0.085	0.013
	Proteobacteria	*Desulfovibrio*	0.009	0.029	0.007	0.007
		*Moraxella*	0.282	0.453	0.039	0.041

Only the taxa which have over 0.01% average relative abundance in at least one of the treatments [BE(+), BE(−), PE(+), PE(−)] were statistically analyzed by LEfSe

BE, bacterial-enriched rumen fluid; PE, protozoal-enriched rumen fluid

*Calculated based on the normalized read count BIOM tables

There were no archaeal phyla and genera differed by either of microbial inoculation treatments. Although not different by microbial inoculation treatments, *Methanocaldococcus* represented averagely over 60% of the total archaeal community of dairy calves (data not shown).

**Fig. 3:**
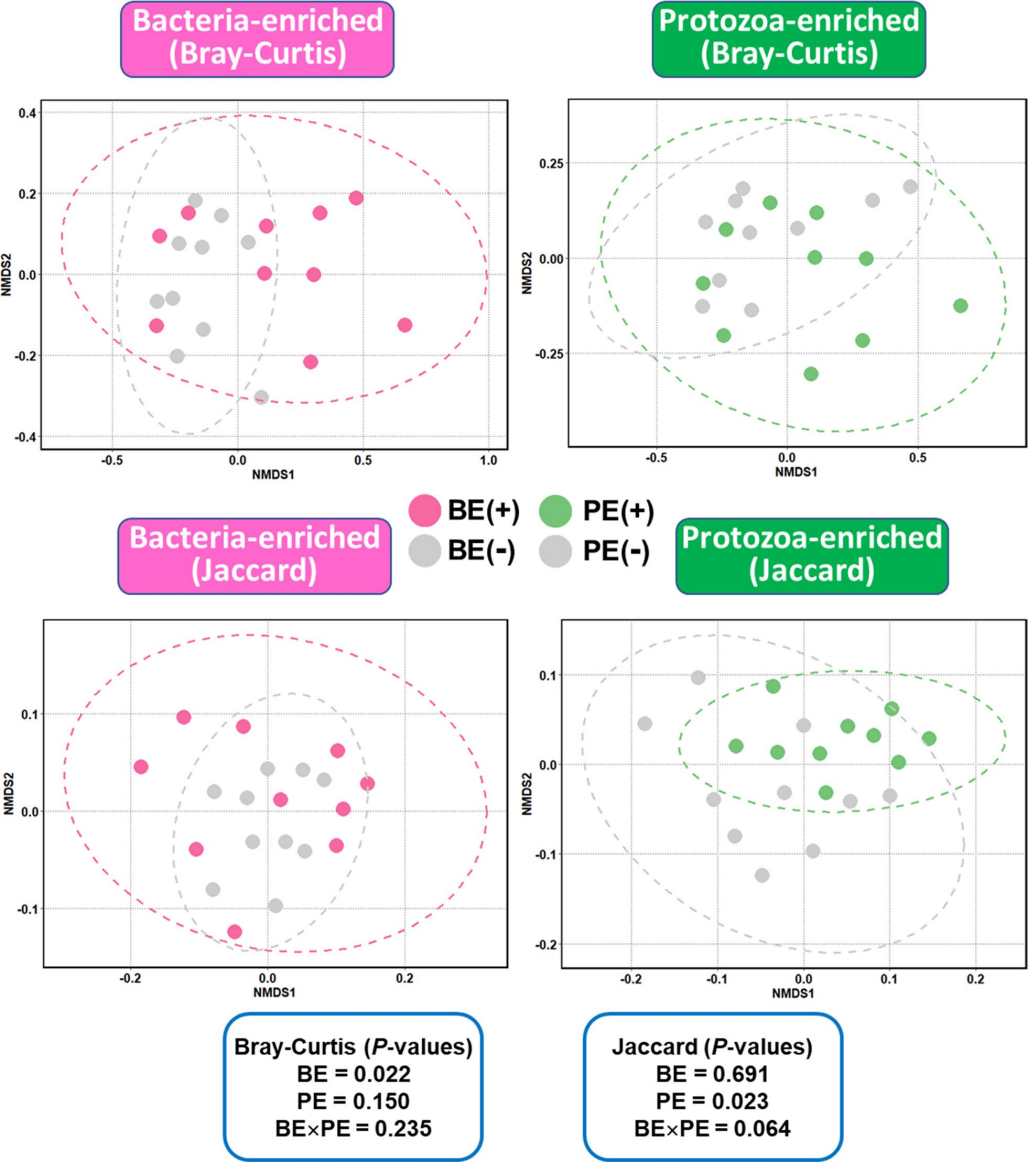
NMDS plot based on Bray–Curtis and Jaccard distance matrices showing overall microbial functional distribution represented by the relative abundance of annotated KEGG modules in the liquid fraction of dairy calves differed by microbial inoculations with two types of inocula (BE and PE). BE, bacterial-enriched rumen fluid; PE, protozoal-enriched rumen fluid

**Fig. 4:**
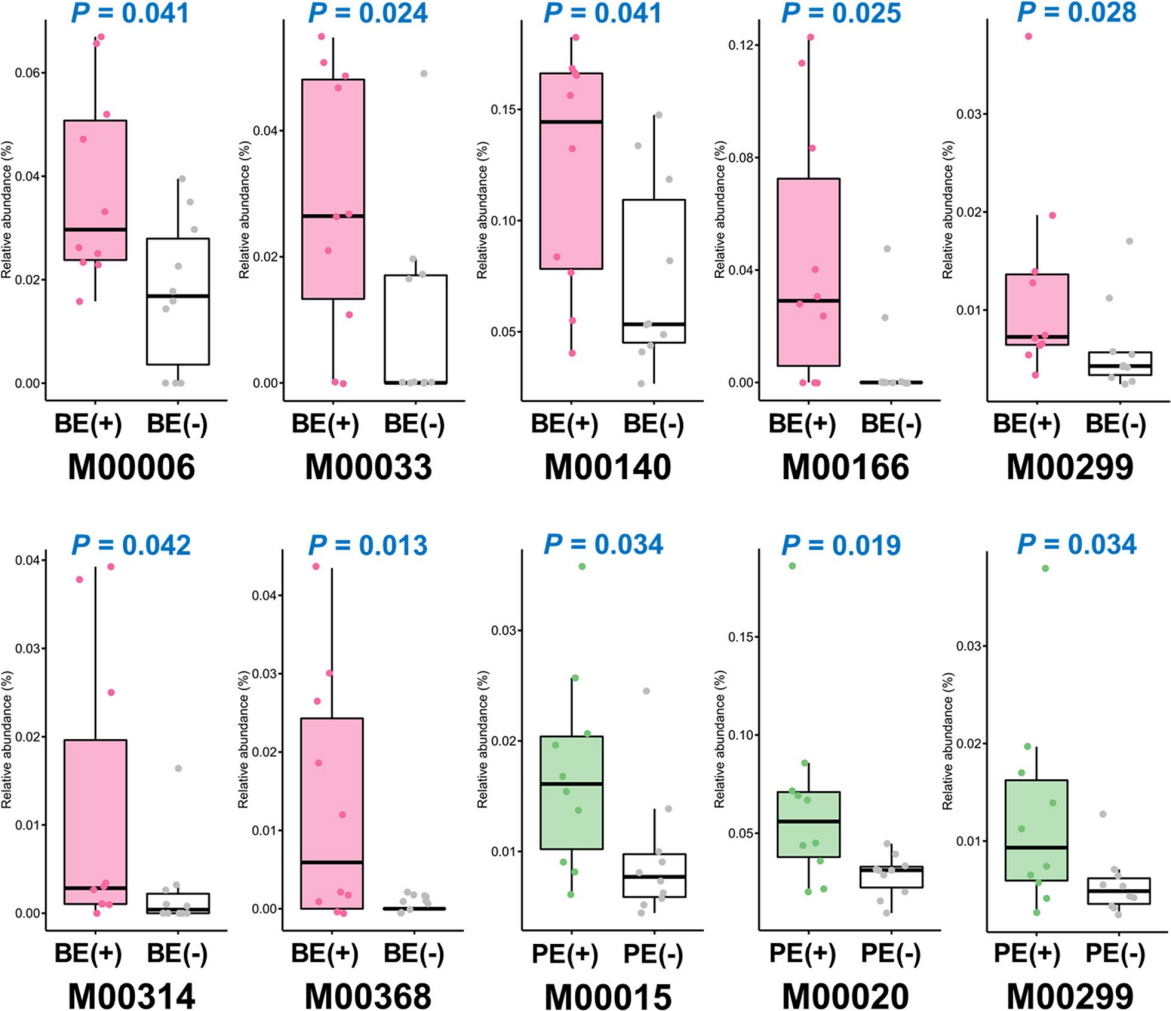
Differentially abundant major KEGG modules by microbial inoculations. Individual data points were jittered on each boxplot. BE, bacterial-enriched rumen fluid; PE, protozoal-enriched rumen fluid

Among the protein sequences deduced from the blastx best hit, an average of 49.22% sequences were mapped to the KEGG orthologs (Additional file 2: Table S2). Based on the KEGG orthologs predicted from the annotation of non-rRNAs, overall microbial functional profiles were affected by microbial inoculations (Fig. 3). BE and PE treatments affected the overall abundance and presence/absence of microbial functions, respectively (*P* < 0.05). For individual relative abundance of KEGG modules, seven KEGG modules [M00006, pentose phosphate pathway, oxidative phase, glucose 6P =  > ribulose 5P (*P* = 0.041); M00033, ectoine biosynthesis, aspartate =  > ectoine (*P* = 0.024); M00140, C1-unit interconversion, prokaryotes (*P* = 0.041); M00166, reductive pentose phosphate cycle, ribulose-5P =  > glyceraldehyde-3P (*P* = 0.025); M00299, spermidine/putrescine transport system (*P* = 0.028); M00314, bacitracin transport system (*P* = 0.042); and M00368, ethylene biosynthesis, methionine =  > ethylene (*P* = 0.013)] were differentially enriched in BE(+) calves rather than BE(−) calves (Fig. 4). The relative abundance of M00015 [Proline biosynthesis, glutamate =  > proline (*P* = 0.034)), M00020 [serine biosynthesis, glycerate-3P =  > serine (*P* = 0.019)], and M00299 [spermidine/putrescine transport system (*P* = 0.034)] were greater in PE(+) calves compared to that of PE(−) calves.

### Adult rumen fluid inoculation effects on the presence/absence of rumen microbial and functional features

**Fig. 5:**
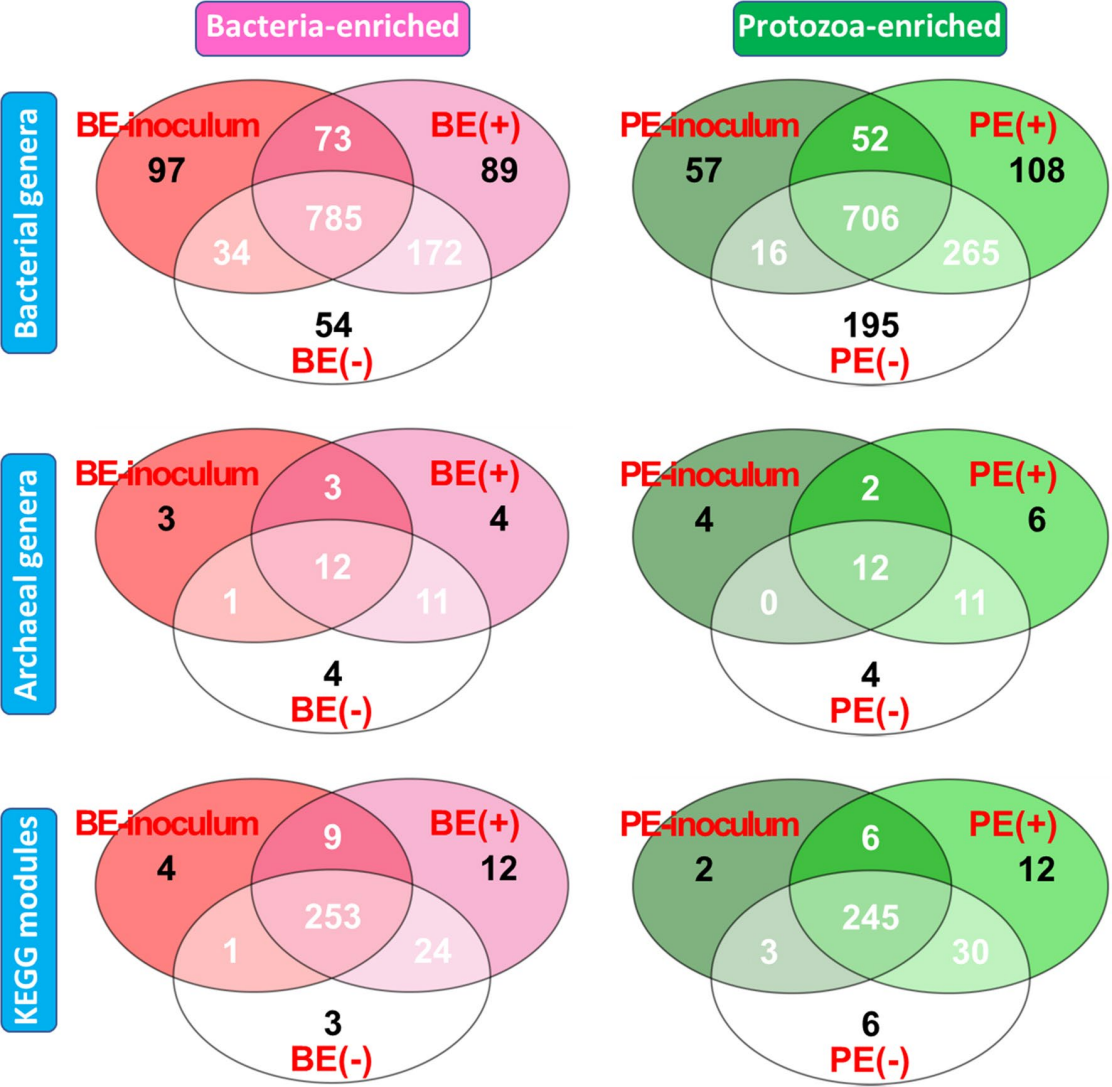
Number of bacterial, archaeal genera and KEGG modules which were differentially observed by microbial inoculations. The number of shared or exclusively found genera or functional features among the corresponded microbial inocula, inocula-treated and controls were shown in Venn diagram. BE, bacterial-enriched rumen fluid; PE, protozoal-enriched rumen fluid

The Venn diagrams showed the number of shared or exclusive bacterial and archaeal taxa at genus level and KEGG modules between microbial inoculation treatments with their corresponding inocula (Fig. 5). Among the number of classified archaeal genera, 31.58% of total archaeal genera were shared among the BE-inoculum, BE(+), and BE(−) and 28.95% of total archaeal genera were exclusively found in one of the three conditions. Among the number of classified bacterial genera, 60.20% of total bacterial genera were shared among the three conditions by BE inoculation and 18.40% were exclusively found in one of the three conditions. Among 306 KEGG modules, 253 were shared by the three conditions by BE inoculation and only 19 KEGG modules were exclusively found in one of the three conditions. By PE inoculation and its inoculum, 50.46% and 30.77% of bacterial and archaeal genera were shared among PE-inoculum, PE(+), and PE(−), respectively. Otherwise, 25.73% and 35.90% of bacterial and archaeal genera were exclusively found either in PE-inoculum, PE(+), and PE(−), respectively. Among the identified 304 KEGG modules, 245 were shared by the three conditions by PE inoculation and only 20 KEGG modules were exclusively found in one of the three conditions. Both bacterial and archaeal genera, and their identified microbial functions represented by KEGG modules potentially transferred from inocula to inocula-treated calves only occupied minor abundance in both the inoculations (< 0.0001% of bacterial and archaeal abundances whereas < 0.004% of KEGG modules). In both of the inoculations, the inocula shared a numerically greater number of archaeal and bacterial genera, and KEGG modules with inoculum-treated calves compared to that of non-treated calves.

### Changes in the co-occurrence or mutual-exclusive microbiome networks of rumen after adult rumen fluid inoculation

**Fig. 6:**
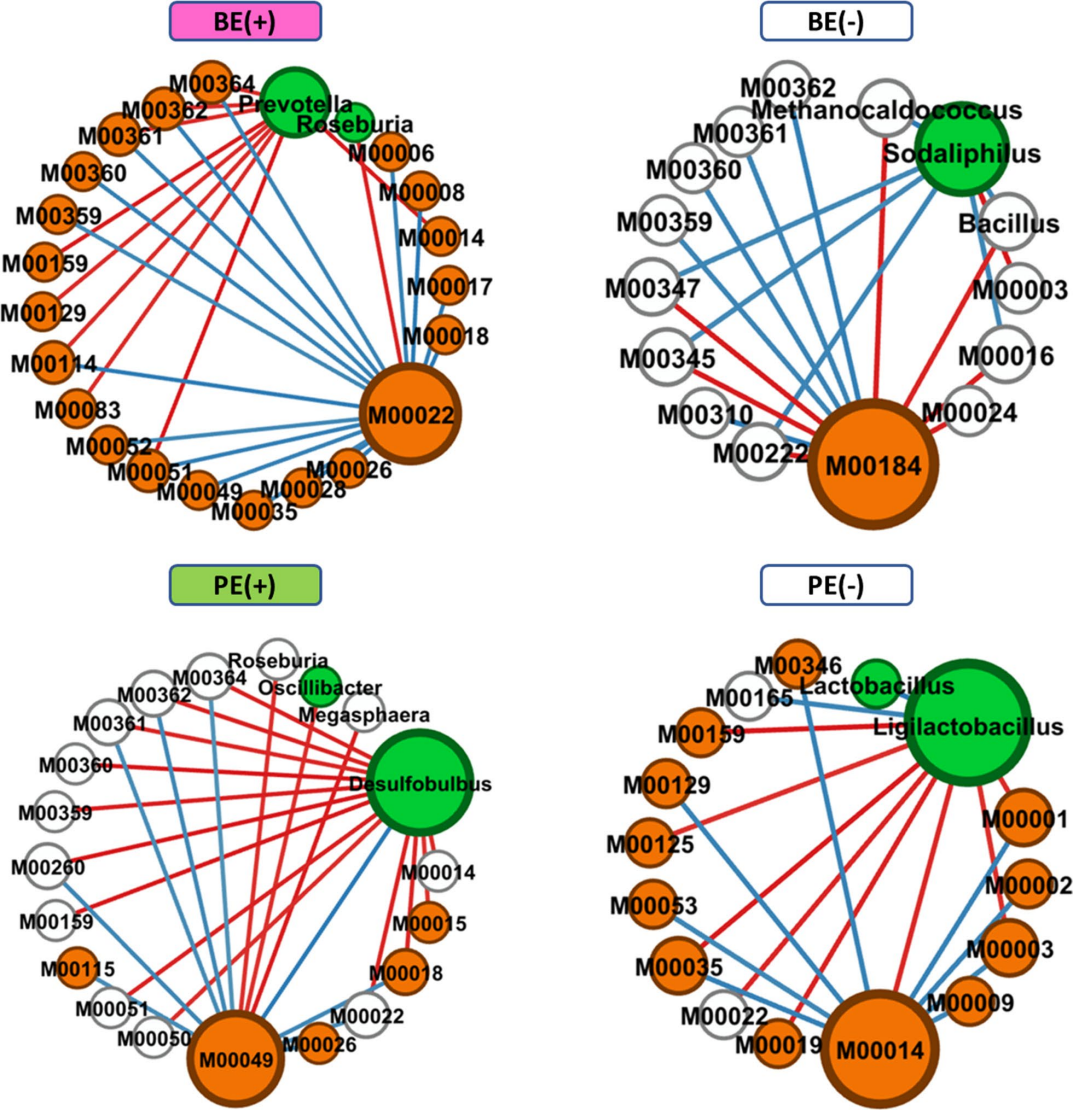
Circular type networks interacted with keystone nodes (either microbial or functional) at each microbial inoculation treatment. Exclusive node was denoted by blue for archaeal, green for bacterial and orange for the functional features. Edge color represents co-occurrence (blue) or mutual exclusive (red) interactions. Edge thickness was adjusted based on the absolute value of the correlation coefficients of each interaction. BE, bacterial-enriched rumen fluid; PE, protozoal-enriched rumen fluid

The complexed co-occurrence and mutual exclusive interactions among the abundance of major bacterial and archaeal genera and KEGG modules differed by microbial inoculations (Additional file 5: Fig. S4). By BE, 99 (70 KEGG modules, four archaeal genera, and 25 bacterial genera) and 22 (13 KEGG modules, one archaeal genus, and eight bacterial genera) nodes with 260 and 123 significant interactions were exclusively found in BE(+) and BE(−) treated calves, respectively (Additional file 5: Fig. S4). A total of 37 (27 KEGG modules, two archaeal genera, and eight bacterial genera) and 71 (48 KEGG modules, two archaeal genera, and 21 bacterial genera) nodes with 199 and 194 significant interactions were exclusively found in PE(+) and PE(−) calves, respectively. Based on the two centrality measurements within the exclusive interactions in each inoculation treatment, *Prevotella* and M00022 (Shikimate pathway, phosphoenolpyruvate + erythrose-4P =  > chorismate) for BE(+) calves whereas *Sodaliphilus* and M00184 (RNA polymerase, archaea) for BE(−) calves were identified as keystone microbial and functional nodes by BE inoculation (Table 3). By PE inoculation, *Desulfobulbus* and M00049 (Adenine ribonucleotide biosynthesis, IMP =  > ADP,ATP) for PE(+) calves whereas *Ligilactobacillus* and M00014 [Glucuronate pathway (uronate pathway)) for PE(−) were the keystone nodes. The interactions centered on those keystone nodes were further selected and visualized in Fig. 6.

**Table 3:** Exclusive network statistics by BE or PE

Network measurements	Microbial inoculations			
	BE(+)	BE(−)	PE(+)	PE(−)
Total nodes	137	106	137	128
Total edges	260	123	199	194
Positive	193	77	132	136
Negative	67	46	67	58
Positive (%)	74.231	62.602	66.332	70.103
Negative (%)	25.769	37.398	33.668	29.897
No. of exclusive nodes	MD: 70; Arc: 4; Bac: 25	MD: 13; Arc: 1; Bac: 8	MD: 27; Arc: 2; Bac: 8	MD: 48; Arc: 2; Bac: 21
Abundance of exclusive node (%)	MD: 6.462% Arc: 24.597% Bac: 21.120%	MD: 0.418% Arc: 0.549% Bac: 1.569%	MD: 0.857% Arc: 9.485% Bac: 1.441%	MD: 5.084% Arc: 67.380% Bac: 14.901%
Network diameter	16	13	16	12
Graph density	0.028	0.022	0.021	0.024
Modularity	0.675	0.815	0.744	0.706
No. of communities	17	16	22	22
Average clustering coefficient	0.301	0.117	0.306	0.268
Average path length	5.843	5.741	5.810	4.627
Best 'Authority' node	M00022 *Prevotella*	M00184 *Sodaliphilus*	M00049 *Desulfobulbus*	M00014 *Ligilactobacillus*
Best 'Eigenvector centrality' node	M00022 *Prevotella*	M00184 *Sodaliphilus*	M00049 *Desulfobulbus*	M00014 *Ligilactobacillus*

BE, bacterial-enriched rumen fluid; PE, protozoal-enriched rumen fluid; MD, KEGG modules; Arc, archaeal genera; Bac, bacterial genera

### Differed CAZymes profiles by microbial inoculations

A total of 186 and 163 CAZymes, belonging to auxiliary activities, carbohydrate-binding modules (CBM), carbohydrate esterases (CE), glycosyl hydrolases (GH), glycosyl transferases (GT), and polysaccharide lyases (PL), were annotated from the BE and PE inoculum, respectively, and there were no differentially abundant CAZymes between BE and PE inoculum (data not shown).

GH19, GT84, and PL14 were enriched in BE(+) calves whereas the dominance of CE14 and GH89 was found in BE(−) calves compared to their counterparts (Table 4). The predominance of 11 CAZymes were divided by PE inoculation. However, dominance of those CAZymes in each type of microbial inoculation were not observed in their corresponded inoculums.

**Table 4:** Differentially abundant CAZymes by microbial inoculations

Dominance	CAZymes	Relative abundance (%)^*^		SEM	*P*-value	Known activities
		BE(+)	BE(−)			
BE(+)	GH19	0.010	0	0.003	0.031	chitinase (EC 3.2.1.14)
	GT84	0.053	0.004	0.013	0.009	cyclic β-1,2-glucan synthase (EC 2.4.1.-)
	PL14	0.067	0.013	0.011	0.010	alginate lyase (EC 4.2.2.3)
BE(−)	CE14	0.195	0.310	0.032	0.041	N-acetyl-1-D-myo-inosityl-2-amino-2-deoxy-α-D-glucopyranoside deacetylase
	GH89	0.147	0.242	0.026	0.023	α-N-acetylglucosaminidase

Dominance	CAZymes	Relative abundance (%)		SEM	*P*-value	Known activities
		PE(+)	PE(−)			
PE(+)	GH16	0.314	0.182	0.038	0.041	xyloglucan:xyloglucosyltransferase (EC 2.4.1.207)
	GH39	0.363	0.253	0.056	0.028	β-xylosidase (EC 3.2.1.37)
	GH142	0.125	0.054	0.017	0.023	β-L-arabinofuranosidase (EC 3.2.1.185)
	PL10	0.121	0.032	0.017	0.006	pectate lyase (EC 4.2.2.2)
	PL33	0.051	0.015	0.008	0.006	hyaluronate lyase (EC 4.2.2.1)
PE(−)	CBM2	0.011	0.098	0.019	0.033	cellulose-binding domain
	CE11	0.662	0.991	0.061	0.007	UDP-3–0-acyl N-acetylglucosamine deacetylase (EC 3.5.1.108)
	GH92	0.574	0.959	0.082	0.010	mannosyl-oligosaccharide α-1,2-mannosidase (EC 3.2.1.113)
	GH130	0.416	0.864	0.084	0.008	β-1,4-mannosylglucose phosphorylase (EC 2.4.1.281)
	GH133	0.861	1.161	0.067	0.023	amylo-α-1,6-glucosidase (EC 3.2.1.33)
	GT4	2.064	2.602	0.097	0.005	sucrose synthase (EC 2.4.1.13)

Only the CAZymes which have over 0.01% average relative abundance in at least one of the treatments [BE(+), BE(−), PE(+), PE(−)] were statistically analyzed by LEfSe

BE, bacterial-enriched rumen fluid; PE, protozoal-enriched rumen fluid

*Calculated based on the normalized CAZymes count tables

### Major active bacterial and archaeal genera significantly correlated to the various fermentation and animal measurements

**Fig. 7:**
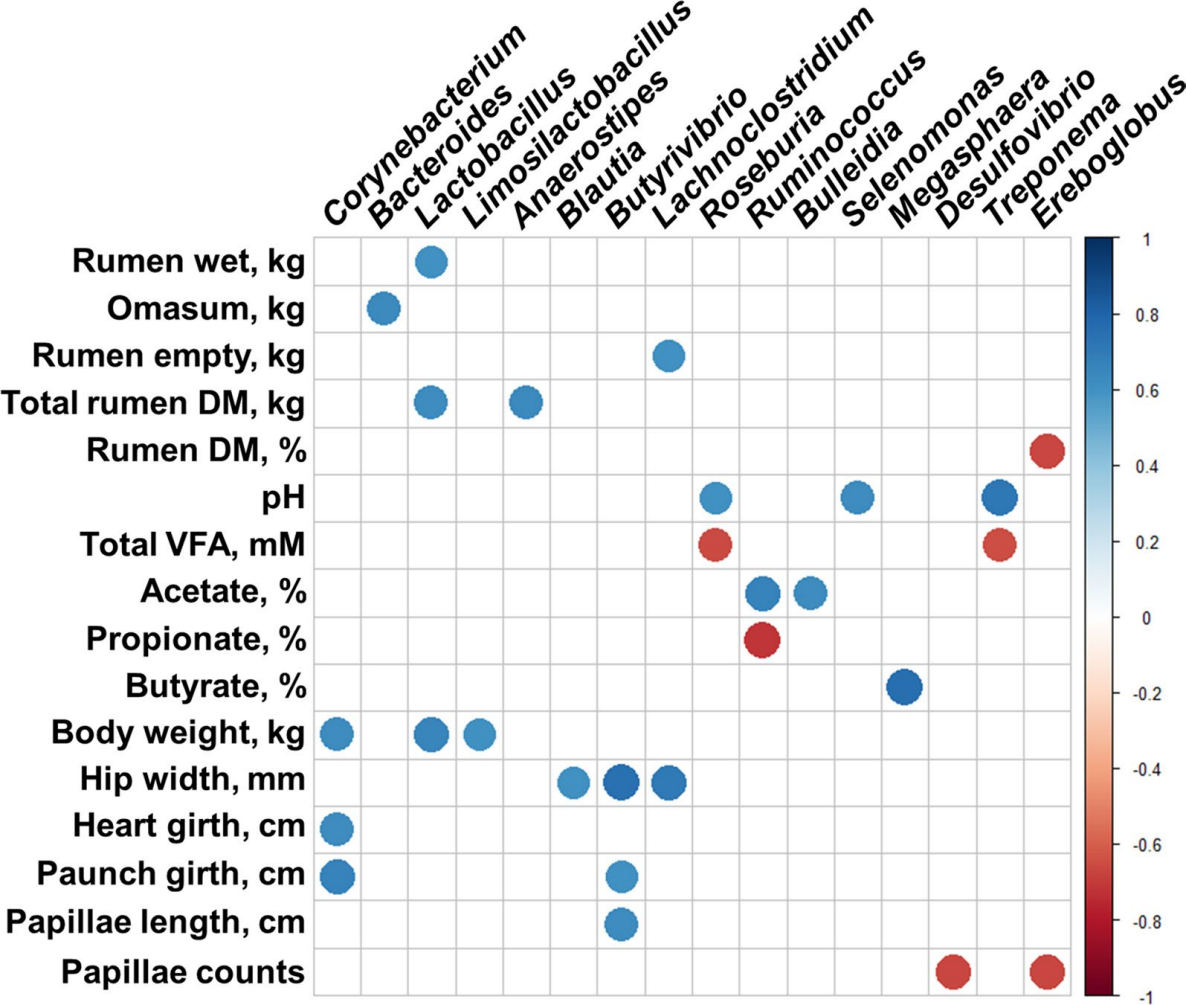
Correlation between animal measurements with the relative abundance of major active bacterial and archaeal genera (occupying over 0.01% average relative abundance in at least one of the treatments). Only strong Pearson correlation coefficients (*P* ≤ 0.05; |*r*|≥ 0.6) were shown on the plot. The correlation coefficients were based both on the size and intensity of the color based on the color key on the right side

Correlation analysis between the relative abundance of major active bacterial and archaeal genera and rumen fermentation or animal performance measurements showed various significant associations (correlation coefficient, |*r*|≥ 0.6; *P* < 0.05) (Fig. 7). Particularly for the different measurements in our previous study [15], three bacterial genera (*Corynebacterium*, *Lactobacillus*, and *Limosilactobacillus*) were positively correlated with body weight. *Butyrivibrio* was positively correlated to three of the animal measurements (hip width, paunch girth, and papillae length). *Treponema* was negatively correlated to total volatile fatty acids (VFA) concentration but positively correlated to ruminal pH. A known butyrate producer, *Megasphaera* was positively correlated to the molar proportion of butyrate which were significantly higher in PE(−) calves than that of PE(+) calves. Even though the measurements were not differed by microbial inoculations, *Desulfovibrio* and *Ereboglobus* were negatively correlated to papillae counts from dairy calves.

## Discussion

Dairy cattle organic matter utilization is inextricably linked with the microbes resident in the rumen. While the microbial community composition is influenced by the environment that the calves inhabit in early life, modifying the microbes to affect a desired productive phenotype could result in benefits for farmers. The established microbial community composition has proved to be resilient toward intended manipulation [1, 2, 38]. For this reason, we and others [11–15] have begun evaluating the possibility of intervening in microbial establishment to direct the community composition by early life inoculation. In this study, the microbial and functional shifts by pre-weaning microbial inoculations (BE and PE) were analyzed by RNA-seq which enables the investigation of active transcript features in the rumen microbiome of post-weaned dairy calves.

### Differential bacterial taxa between RNA-seq and 16S amplicon-seq

Among the shared major phyla and genera between two analytic pipelines, the relative abundances of Elusimicrobia, Fibrobacteres, Planctomycetes, Spirochaetes, and *Butyricimonas*, *Fibrobacter*, *Oscillibacter*, *Selenomonas*, *Treponema* were at least tenfold higher in RNA-seq compared to 16S amplicon-seq and those taxa might be metabolically active at least in post-weaned dairy calves. In a previous study comparing three different microbiota datasets in beef steers [17], the relative abundance of Bacteroidetes, represented by the most abundant ruminal genus, *Prevotella*, was greater in the 16S amplicon-seq dataset compared to that of RNA-seq. Although *Prevotella* is the predominant ruminal bacterial genus in a variety of ruminal conditions [39–41], its relative abundance was 5.41-fold lower in RNA-seq datasets compared to that of 16S amplicon-seq in this study. Based on these results, we speculate that their metabolic contribution to the overall rumen microbiome might have been overstated. However, longitudinal study based on the RNA-seq pipeline will be necessary to further evaluate their fluctuation patterns in different dietary conditions particularly in adult dairy cattle.

The lack of differential relative abundance of Firmicutes between two analytic pipelines was consistent with previous comparison. However, the result on the abundance of Proteobacteria between the two methods was contrasting. In this study, the abundance of Proteobacteria represented by four genera, *Desulfobulbus*, *Desulfovibrio*, *Campylobacter*, and *Succinivibrio*, was more predominant in RNA-seq datasets (0.93% in RNA-seq vs. 0.21% in 16S amplicon-seq). It is plausible that differing diets provided to both dairy and beef cattle differentially affected the activity of a variety of bacterial taxa. Furthermore, predominance of several fibrolytic bacterial genera (*e.g*., *Fibrobacter*, *Clostridium*, and *Ruminococcus*) in RNA-seq datasets could be used as a potential barometer to evaluate fibrolytic activities in dairy cattle using metatranscriptomic approaches. Additionally, the taxa uniquely identified by RNA-seq, if those taxa have not been widely studied in the rumen, would fill the blind spots of 16S-based methods.

### Microbial inoculations affect the presence and absence of taxa distributions

The active bacterial and archaeal genera exclusively detected in either the inoculum or inoculum-treated calves were minor taxa based on the relative abundance cutoff. This reveals that an active core of microbiota in the rumen liquid were shared among adult rumen fluid, microbial inoculum treated and non-treated calves. This may have resulted in similar animal phenotypes and fermentation by microbial inoculations.

### Differential bacterial taxa by microbial inoculations

Proteobacteria was the only phylum altered by microbial inoculations, specifically by PE. This phylum has been known as major bacterial group in ruminants and particularly dominant in young ruminants [4, 41]. Lower abundance of this phylum by PE might be directly triggered by inoculation of adult rumen fluid which was predominated by Bacteroidetes and Firmicutes. Or rumen fluid inoculation facilitated the transition of rumen microbial community of dairy calves to that of less proteobacterial-dominated which has been noticed by longitudinal observation in young ruminants [4, 5].

*Bifidobacterium* is not usually considered as major genus in adult rumen [42] and the abundance of this genus in BE(+) might be lowered by the establishment of normal microflora transferred from adult rumen fluid [9]. Among the bacterial genera predominant in BE(−) calves, *Anaerostipes* might contribute to butyrate production [43] in the rumen. However, there were no phenotypic differences found in their predominance between BE(−) and BE(+) calves.

PE(+) calves were dominant in *Eubacterium* and this genus containing the species of cellulose- and hemicellulose-degrading culturable rumen bacteria [31] and has been isolated from the bovine rumen [44]. Its predominance indicates an increase in fiber digestion. *Desulfobulbus* produces acetate as the major fermentation products of mono- and disaccharide fermentation [45]. Their predominance in PE(+) calves might affect the higher total ruminal VFA concentration compared to that of the PE(−) calves. A greater relative abundance of two butyrate producing bacteria might have resulted in the increase of the butyrate production in the PE(−) calves expecting to show better growth of rumen epithelial cells, papillae and development of the rumen [3, 46]. However, as noticed in BE treatment, no phenotypic differences were corresponded to the greater butyrate molar proportion in the PE(−) calves.

*Roseburia* is a strong butyrate producer due to its ability to ferment starch [47]. The lactate-utilizing bacteria, *Megasphaera*, dominant in PE(−) calves also contributed to the greater butyrate molar proportion by converting lactate to butyrate [48, 49]. Previous defaunation studies demonstrated that ruminal defaunation was negatively associated with butyrate molar proportion [50]. Only one calf in this study harbored holotrichs that are able to produce butyrate [51], whereas butyrate production by entodiniomorphs, the predominant type of ruminal protozoa, is not well-known. In addition to the negative correlation between NDF digestibility and butyrate proportions in defaunated animals [50], differed butyrate proportion by PE inoculation was related to the fiber digestibility and the composition of the metabolically active microbes varied in each treatment.

Although, there were no differed archaeal taxa by microbial inoculations in this study, the overwhelming abundance of *Methanocaldococcus* in the active rumen microbiome of dairy calves in this study, in addition to the observance of its predominance in rumen wall by using metatranscriptome sequencing [52], needs to be further studied in terms of the actual contribution by this hydrogenotrophic methanogen to the overall rumen methane production.

### Functional distribution

KEGG orthologs were predicted from the putative mRNAs of ruminal microbes. We extracted functional annotations from the blastx best hit outputs since the other metabolic pathway profiling tool (*e.g.,* HUMAnN2) only mapped 1.60% of the reads to the known functions (data not shown). This could be due to the limited availability of functional genomic information of ruminal microbes [53].

For DNA-based methods, the differential microbial community or individual taxa abundances did not alter the microbial functions due to the functional redundancies among rumen microbes [54]. However, we observed that the overall microbial functional distribution was affected quantitatively by BE inoculation and qualitatively by PE inoculation. Even though the adult rumen fluid inoculation didn’t change the overall ruminal bacterial communities, population shifts of the active microbiome and newly transferred microbial functions might result in the slightly altered animal performance and fermentations profiles of dairy calves. More distinguishable phenotypic changes by PE rather than by BE might result from the establishment of rumen protozoa, which affects a variety of microbial interactions and their symbiotic relationships.

### Differential individual functional feature abundances

Among the analyzed fermentation characteristics, only ruminal pH was tended to be lower in BE(+) calves compared to that of BE(−) calves [15]. Two KEGG modules (M00006 and M00166) from BE(+) calves that participate in the formation of glyceraldehyde-3-phostphate via conversion of glucose to pyruvate may have contributed to a decrease in pH through the increased production of hydrogen [55].

The relative abundance of two amino acids biosynthesis modules (M00015 and M00020) was greater in PE(+) calves than that of PE(−) calves but this differential functional abundance did not result in increased total free amino acid concentrations and molar proportions of isobutyrate in PE(+) calves.

Spermidine and putrescine are synthesized from arginine [56] and possibly related to handling oxidative stress in the rumen [57]. The transport system of those polyamines was differentially abundant by both the BE and PE treatment and this is in agreement with the result of previous repeated adult rumen fluid treatment study with male calves [8].

### Exclusive microbial and functional networks by microbial inoculations

The overall interactions within the rumen microbiome, which is represented by the abundance of major bacterial and archaeal genera and KEGG modules, were compared between microbial inoculation treatments. Shikimate pathway module (M00022) was the keystone function based on the centrality measurements within the exclusive networks in BE(+) calves. Together with the differentially enriched module related to pentose phosphate pathway (M00006) in BE(+) calves, shikimate pathway links between carbohydrate metabolism and aromatic amino acids biosynthesis and is a strong indicator of anabolic processes in the rumen microbiome [58, 59]. Although, none of the exclusive positive interactions were found with phenylalanine, tyrosine and tryptophan biosynthesis pathway modules, shikimate pathway positively associated with carbohydrate pathways and many of biosynthesis pathways including vitamin, ribonucleotides, aminoacyl-tRNA, nucleotide sugars, and isoprenoid. This might be associated with the greater microbial protein synthesis and its growth triggered by BE inoculation, but they did not result in better fermentative and animal performances for BE(+) calves. Whereas, keystone bacterial genus, *Prevotella* showed negative associations with many of aforementioned biosynthesis pathway modules. Negative associations directly or indirectly related to the biosynthesis pathways (M00083, M00114, M00129, M00159, M00361, M00362, and M00364) with this keystone bacterial node implied the potential inefficient biosynthetic processes in *Prevotella*-dominant rumen microbiome of dairy calves.

In BE(−) calves, M00184 and *Sodaliphilus* were the keystone nodes within the exclusive network. The keystone nodes showed opposite associations particularly to the hydrogenotrophic methanogenesis pathway (M00347) and the abundance of *Methanocaldococcus*. M00184 is related to archaeal function [60] but its negative association with archaeal genus and methanogenesis pathway module need to be further studied in the context of archaeal transcription mechanism. *Sodaliphilus*, which was firstly described within pig microbiome [61], has been poorly characterized within rumen microbiome. Since its genome lacks genes encoding multiple glycolytic proteins [61] and hydrogenase in addition to its requirement of co-cultivation for better growth, the exclusive positive interaction with hydrogenotrophic methane production pathway might be indirect. Methane production measurements will be required to demonstrate the capacity of this exclusive networks in BE(−) calves.

Keystone nodes (M00049 and *Desulfobulbus*) in the exclusive network of PE(+) calves were positively associated each other and this interaction need to be understood within protozoa-harboring rumen microbiome. Purine metabolism is related to the cell growth and death [62], and this might be associated with bacterial predation activity of rumen protozoa inoculated by PE. In addition to its differential abundance in PE(+) calves, sulfate-reducing *Desulfobulbus* and its association with rumen protozoa is yet to be determined. Negative association with protein synthesis related pathway modules might be also related to having rumen protozoa particularly dominated by bacterivory entodiniomorphs [63].

Glucuronate pathway module (M00014) was the keystone functional module in the PE(−) calves positively interacted with many of the carbohydrate metabolisms (e.g., glycolysis, gluconeogenesis, citrate cycle) and formaldehyde assimilation module (M00346), which drives by methanotrophic bacteria to oxidize methane and methanol to formaldehyde, producing more hydrolytic products including pyruvate and methane [64]. However, these exclusive associations did not lead to fermentative responses beneficial to PE(−) calves. In addition to finding a greater number of enriched CAZymes in PE(−) calves compared to those of PE(+), the microbiome of PE(−) calves was associated with more complexed carbohydrate metabolism which usually linked to the inefficient utilization of feed by host animals [65, 66]. *Ligilactobacillus* was a keystone bacterial genus in the exclusive network of PE(−) calves. This genus is positively associated with another lactic acid bacteria, *Lactobacillus*. The occurrence of those probiotic bacterial genera, which enhance the gut health [67], might contribute to numerically better incidence of health measurements in PE(−) calves.

### Active bacterial genera correlated with fermentation and animal measurements

Lactic acid bacteria, represented by the strains of *Lactobacillus* spp., has been widely used as probiotics to enhance the growth and health of young ruminants by preventing the colonization of pathogenic bacteria and boosting immune response [68]. Although the ruminal abundance of *Lactobacillus* is only numerically higher in inocula-treated calves, strong correlation between its abundance and body weight is plausible. Limited information is available for *Corynebacterium* but only mixed result was reported on the linkage between its jejunal abundance and feed efficiency of beef steers [69].

Butyrate is able to stimulate rumen papillae growth and development [70, 71]. Even though both the BE and PE didn’t show different papillae length, correlation analyses demonstrated the positive correlation between the relative abundance of major butyrate producer, *Butyrivibrio*, and papillae length together with the hip width and paunch girth in post-weaned dairy calves. The relative abundances of two *Desulfovibrio* and *Ereboglobus* were negatively correlated with papillae counts. Increased abundance of sulfate reducing bacteria has been linked to the decreased integrity of rumen epithelium due to the H_2_S production [72]. *Ereboglobus* is known as pectinolytic bacteria, and its abundance positively correlated to the acetate and propionate production [73] which inversely affected butyrate proportion in the rumen. Those association patterns found in active microbiome of dairy calves analyzed by using RNA-seq was quite different with those found in our previous amplicon sequencing based datasets.

## Conclusions

RNA-seq allows us to understand what is actively transcribed by the rumen microbiome by analyzing both the coding and non-coding RNA species. Compared to our previous results from both the pre- and post-weaned calves analyzed by 16S ampliconseq approaches, differentially abundant, active microbial taxa and functions analyzed by RNA-seq showed reliable connections with different fermentation characteristics and animal development measurements by adult rumen fluid inoculations. Interestingly, both the BE and PE inocula affected the quantitative and qualitative overall microbial functional distributions, respectively without microbial community differences. In addition to the minor transfer of adult, rumen-derived bacterial taxa into dairy calves, active microbial and functional abundances and network differences might be responsible for the fermentation and performance characteristics of dairy calves during and after inoculations. Future research should focus on the contents from the other compartments of the gastrointestinal tracts from a metatranscriptomics approach, which has the potential to better elucidate undetermined biological factors affected by microbial inoculations in dairy calves.

## Supplementary Information

**Additional file 1: Fig. S1**. Analytic workflow of metatranscriptomics used in this study.

**Additional file 2: Table S1**. Calf health assessments. **Table S2**. RNA sequence statistics in inoculated samples. **Table S3**. RNA sequence statistics in inocula. **Table S4**. Alpha diversity measurements of bacterial and archaeal genera in microbial inocula.

**Additional file 3: Fig. S2**. NMDS plot shows the distribution of bacterial genera which were detected by both the 16S amplicon- and RNA-seq. Plots were drawn based on the Bray-Curtis distance matrices and significance was calculated using PERMANOVA test implemented in Vegan.

**Additional file 4: Fig. S3**. Principal coordinates analysis (PCoA) plot based on Bray-Curtis and Jaccard distance matrices representing overall active rumen archaeal microbiota at the genus level in the liquid fraction of dairy calves differed by microbial inoculations with two types of inocula (BE and PE). BE, bacterial-enriched rumen fluid; PE, protozoal-enriched rumen fluid.

**Additional file 5: Fig S4**. Exclusive co-occurrence and mutual exclusion microbial network by microbial inoculations based on the relative abundance of archaeal and bacterial genera and KEGG modules. Exclusive node was denoted by blue for archaeal, green for bacterial and orange for the functional features. Keystone node was marked as asterisks selected based on the authority and eigenvector centrality measurements within each exclusive network. Edge color represents co-occurrence (blue) or mutual exclusive (red) interactions. Edge thickness was adjusted based on the absolute value of the correlation coefficients of each interaction. BE, bacterial-enriched rumen fluid; PE, protozoal-enriched rumen fluid.

## Data Availability

The raw RNA reads generated for this study can be found in NCBI Sequence Read Archive, PRJNA454463.

## Abbreviations

16S amplicon-seq: 16S rRNA gene amplicon sequencing
BE: Bacterial-enriched rumen fluid
CAZymes: Carbohydrate-active enzymes
KAAS: KEGG Automatic Annotation Server
KEGG: Kyoto Encyclopedia of Genes and Genomes
CBM: Carbohydrate-binding modules
CE: Carbohydrate esterases
CoDiNA: Co-expression Differential Network Analysis
GH: Glycosyl hydrolases
GT: Glycosyl transferases
PE: Protozoal-enriched rumen fluid
PERMANOVA: Permutational multivariate analysis of variance
PL: Polysaccharide lyases
RIN: RNA integrity number
SEM: Standard error of the mean
VFA: Volatile fatty acids
