# Supplementary figures and images for: The rumen liquid metatranscriptome of post-weaned dairy calves differed by pre-weaning ruminal administration of differentially-enriched, rumen-derived inocula
Source: Anim Microbiome. 2022 Jan 5;4:4. doi: 10.1186/s42523-021-00142-z (PMC8728904; doi:10.1186/s42523-021-00142-z)

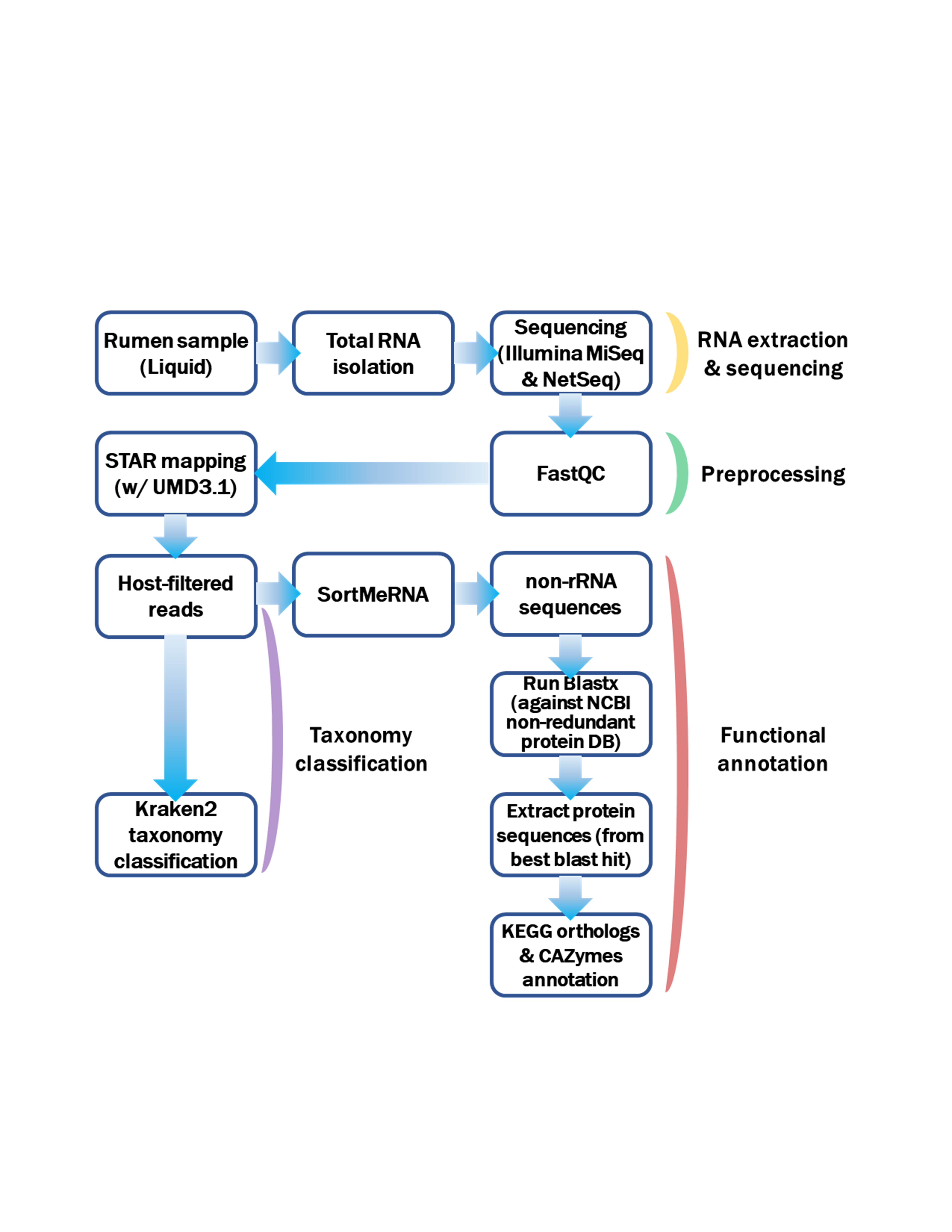

Supplement: Supplementary file 1 — Additional file 1: Fig. S1. Analytic workflow of metatranscriptomics used in this study. [file 42523_2021_142_MOESM1_ESM.tif]

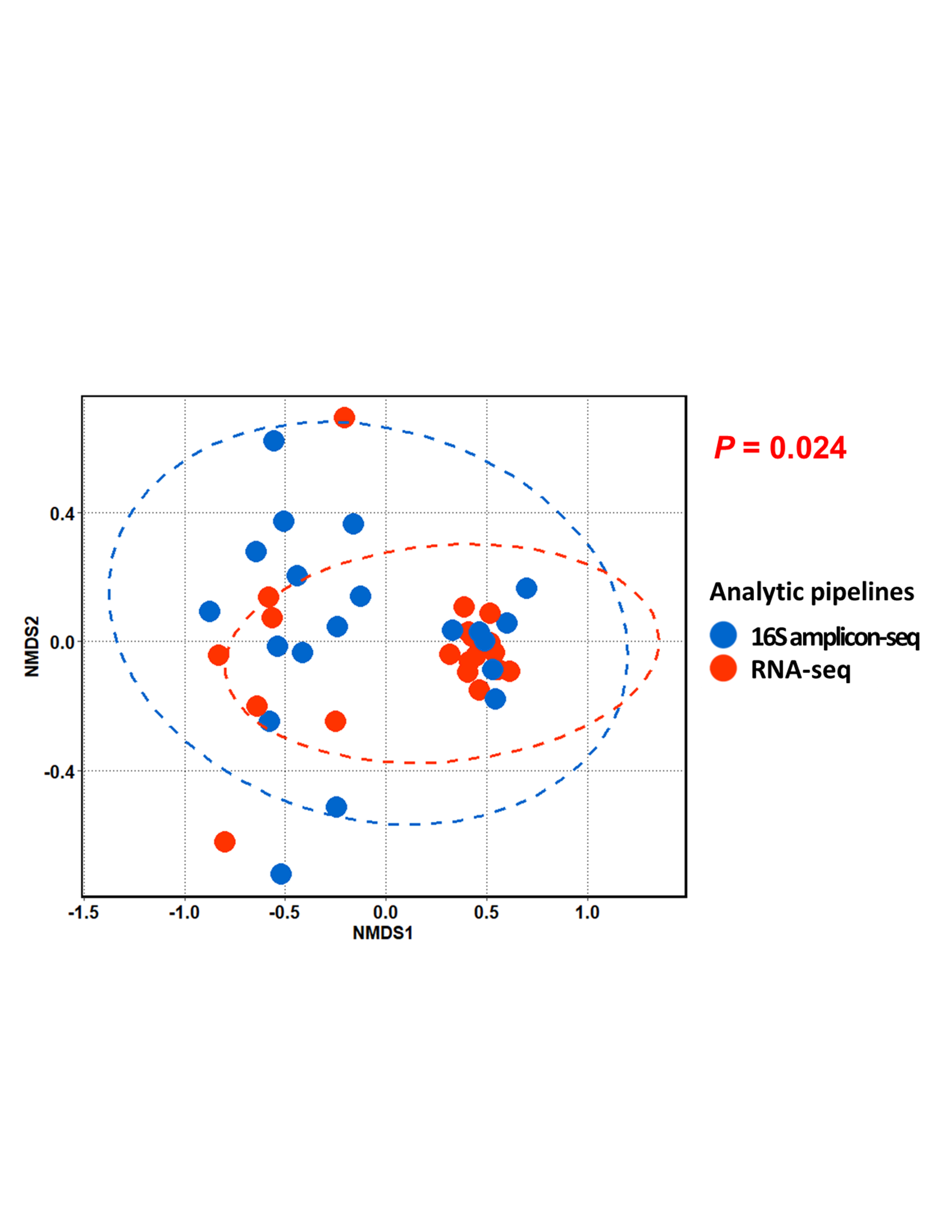

Supplement: Supplementary file 3 — Additional file 3: Fig. S2. NMDS plot shows the distribution of bacterial genera which were detected by both the 16S amplicon- and RNA-seq. Plots were drawn based on the Bray-Curtis distance matrices and significance was calculated using PERMANOVA test implemented in Vegan. [file 42523_2021_142_MOESM3_ESM.tif]

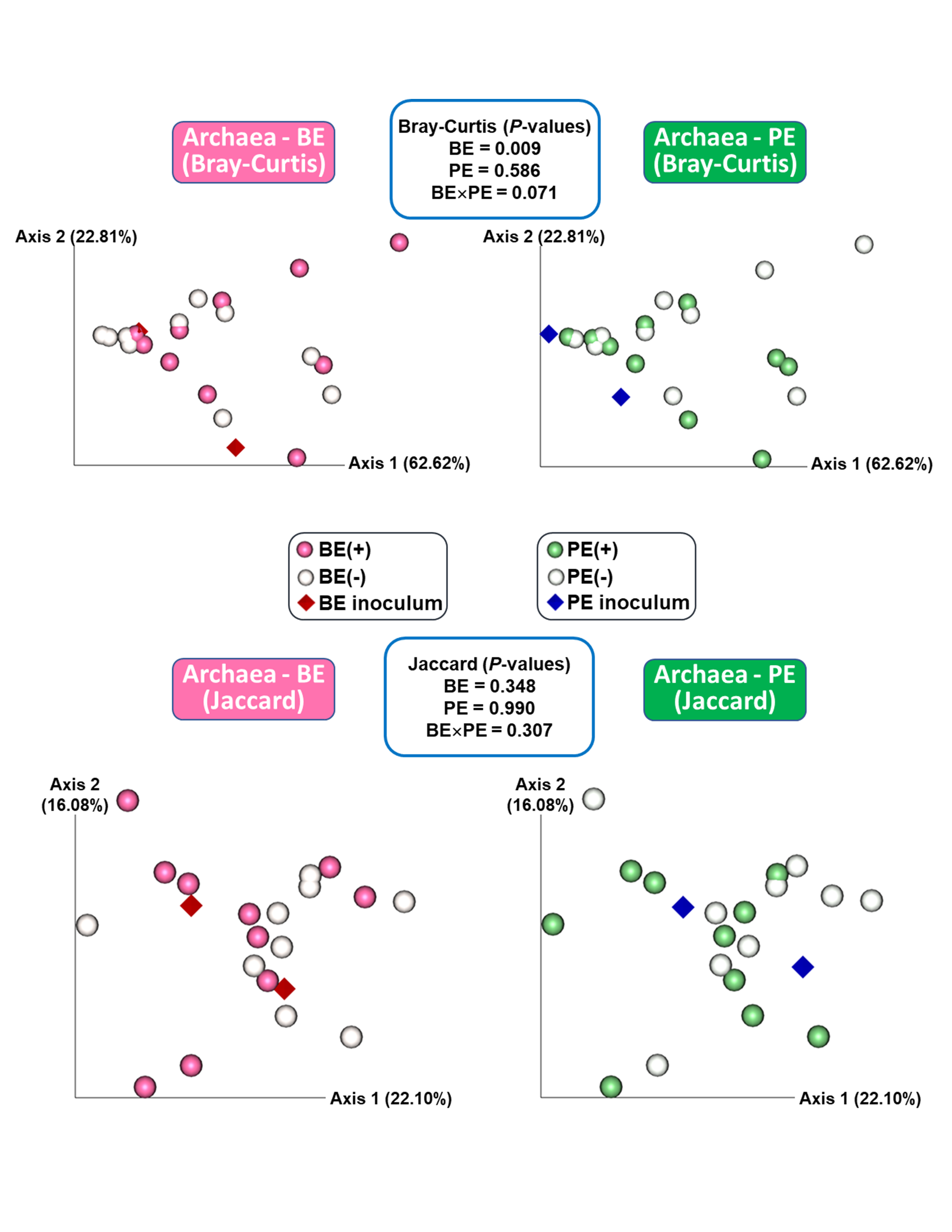

Supplement: Supplementary file 4 — Additional file 4: Fig. S3. Principal coordinates analysis (PCoA) plot based on Bray-Curtis and Jaccard distance matrices representing overall active rumen archaeal microbiota at the genus level in the liquid fraction of dairy calves differed by microbial inoculations with two types of inocula (BE and PE). BE, bacterial-enriched rumen fluid; PE, protozoal-enriched rumen fluid. [file 42523_2021_142_MOESM4_ESM.tif]

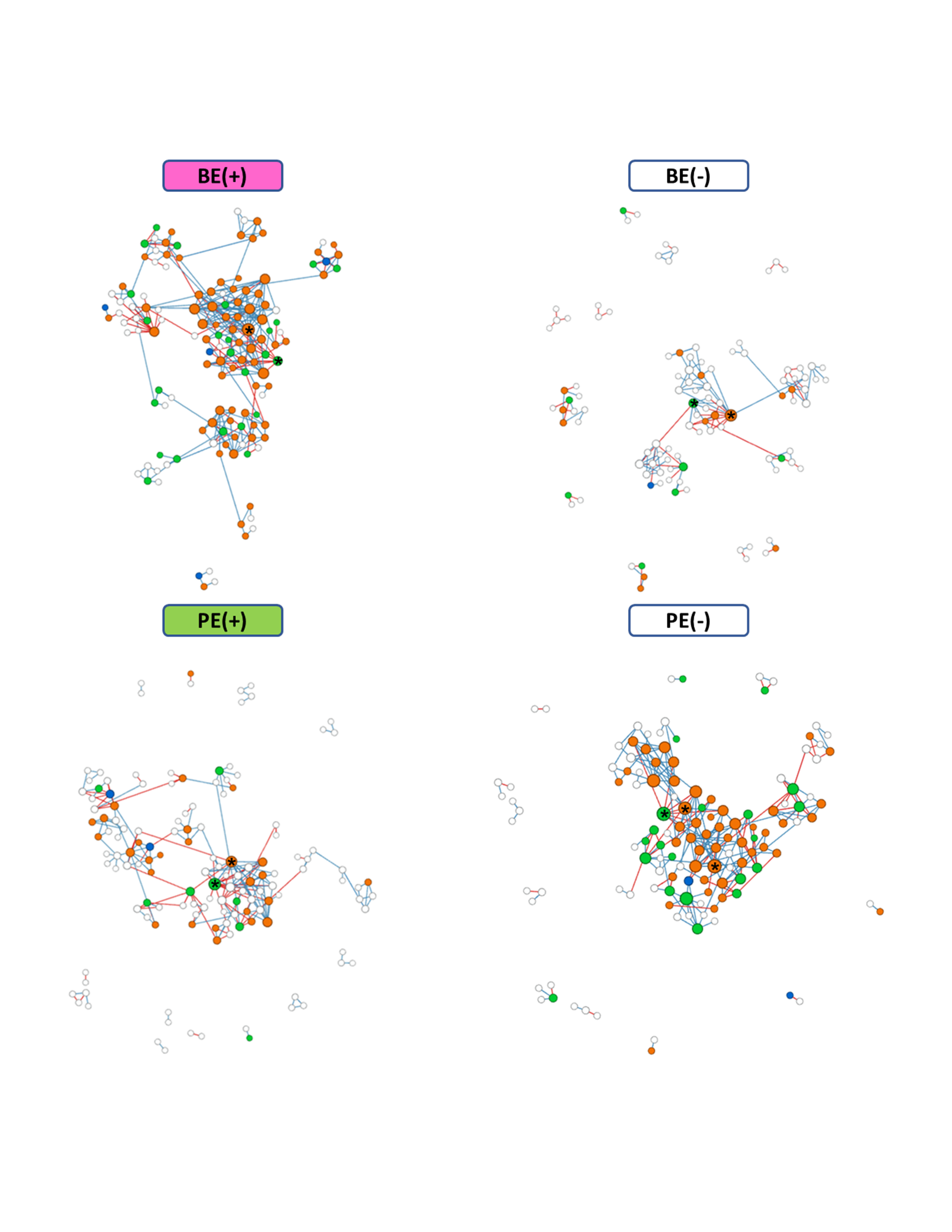

Supplement: Supplementary file 5 — Additional file 5: Fig S4. Exclusive co-occurrence and mutual exclusion microbial network by microbial inoculations based on the relative abundance of archaeal and bacterial genera and KEGG modules. Exclusive node was denoted by blue for archaeal, green for bacterial and orange for the functional features. Keystone node was marked as asterisks selected based on the authority and eigenvector centrality measurements within each exclusive network. Edge color represents co-occurrence (blue) or mutual exclusive (red) interactions. Edge thickness was adjusted based on the absolute value of the correlation coefficients of each interaction. BE, bacterial-enriched rumen fluid; PE, protozoal-enriched rumen fluid. [file 42523_2021_142_MOESM5_ESM.tif]
