# Supplementary material for: The rumen liquid metatranscriptome of post-weaned dairy calves differed by pre-weaning ruminal administration of differentially-enriched, rumen-derived inocula
Source: Anim Microbiome. 2022 Jan 5;4:4. doi: 10.1186/s42523-021-00142-z (PMC8728904; doi:10.1186/s42523-021-00142-z)
Supplement: Supplementary file 2 — Additional file 2: Table S1. Calf health assessments. Table S2. RNA sequence statistics in inoculated samples. Table S3. RNA sequence statistics in inocula. Table S4. Alpha diversity measurements of bacterial and archaeal genera in microbial inocula. [file 42523_2021_142_MOESM2_ESM.docx]

**Table S1.** Calf health assessments.

| **By bacterial-enriched rumen fluid inoculation** | | | | |
| --- | --- | --- | --- | --- |
| Measurements | BE(+) | BE(-) | SEM | *P*-value |
| Rectal Temperature (°C) | 38.92 | 38.75 | 0.133 | NS |
| Nasal discharge | 0 | 0.1 | 0.050 | NS |
| Eye or ear (highest number) | 0.1 | 0.4 | 0.123 | NS |
| Respiratory score | 0.3 | 0.5 | 0.184 | NS |
| Fecal score | 0.4 | 0.2 | 0.147 | NS |
| **By protozoal-enriched rumen fluid inoculation** | | | | |
| Measurements | PE(+) | PE(-) | SEM | *P*-value |
| Rectal Temperature (°C) | 39.06 | 38.61 | 0.133 | NS |
| Nasal discharge | 0.1 | 0 | 0.050 | NS |
| Eye or ear (highest number) | 0.4 | 0.1 | 0.123 | NS |
| Respiratory score | 0.7 | 0.1 | 0.184 | NS |
| Fecal score | 0.5 | 0.1 | 0.147 | NS |

BE, bacterial-enriched rumen fluid.

PE, protozoal-enriched rumen fluid.

NS, no significance (*P* > 0.05).

Rectal temperature was analyzed using SAS with GLIMMIX procedure.

**Table S2.** RNA sequence statistics in inoculated samples.

| Sequence types |  | Average | Bacterial-enriched (BE) | | Protozoal-enriched (PE) | | SEM |
| --- | --- | --- | --- | --- | --- | --- | --- |
|  |  |  | + | - | + | - |  |
| 16S amplicon seqs* | No. of seqs (Rumen liquid) | 26,821 | 28,417 | 25,225 | 27,806 | 25,835 | 1,217 |
|  | No. of seqs (Rumen solid) | 23,285 | 23,808 | 22,763 | 24,594 | 21,977 | 1,739 |
| Total RNA seqs | No. of seqs | 59,153,238 | 59,594,591 | 58,711,885 | 58,237,572 | 60,068,904 | 1,314,352 |
| Host genes | No. of seqs | 171,687 | 151,380 | 191,995 | 159,603 | 183,772 | 8,385 |
|  | %^#^ | 0.291 | 0.254 | 0.327 | 0.276 | 0.306 | 0.013 |
| Host-filtered | No. of seqs | 58,890,367 | 59,353,718 | 58,427,015 | 57,989,246 | 59,791,487 | 1,309,652 |
|  | % | 99.556 | 99.596 | 99.515 | 99.574 | 99.538 | 0.015 |
|  | % of bacteria classified^♣^ | 74.161 | 73.995 | 74.327 | 74.244 | 74.079 | 0.678 |
|  | % of archaea classified^♣^ | 2.647 | 2.500 | 2.793 | 2.605 | 2.688 | 0.268 |
| rRNAs | No. of seqs | 56,567,129 | 57,049,190 | 56,085,068 | 55,825,577 | 57,308,681 | 1,365,863 |
|  | % | 95.628 | 95.729 | 95.526 | 95.858 | 95.405 | 0.576 |
| non-rRNA genes | No. of seqs | 2,323,238 | 2,304,529 | 2,341,947 | 2,163,670 | 2,482,806 | 336,894 |
|  | % | 3.927 | 3.867 | 3.989 | 3.715 | 4.133 | 0.572 |
| Deduced amino acid sequences | No. of seqs | 111,145 | 125,354 | 96,935 | 112,658 | 109,632 | 15,463 |
|  | % | 0.188 | 0.210 | 0.167 | 0.195 | 0.182 | 0.026 |
|  | % of non-rRNAs | 4.784 | 5.439 | 4.139 | 5.207 | 4.416 | 0.322 |
| Annotated  KEGG orthologs | No. of seqs | 52,948 | 56,677 | 49,219 | 52,131 | 53,766 | 6,808 |
|  | % | 0.090 | 0.095 | 0.085 | 0.091 | 0.089 | 0.011 |
|  | % of non-rRNAs | 2.279 | 2.459 | 2.102 | 2.409 | 2.166 | 0.101 |

*, Quality-filtered (>Q25) 16S amplicon sequences were used in our previous study [1].

#, percentage of total RNA sequences.

♣, percentage of host-filtered sequences.

**Table S3.** RNA sequence statistics in inocula.

| Sequence types |  | Average | Bacteria-enriched inoculum | Protozoa-enriched inoculum | SEM |
| --- | --- | --- | --- | --- | --- |
| 16S amplicon seqs* | No. of seqs | 21,938 | 20,822 | 23,055 | 1,162 |
| Total RNA seqs | No. of seqs | 51,733,503 | 52,119,020 | 51,347,987 | 5,150,354 |
| Host genes | No. of seqs | 963,121 | 887,894 | 1,038,348 | 420,892 |
|  | %^#^ | 1.680 | 1.583 | 1.776 | 0.653 |
| Host-filtered | No. of seqs | 50,697,933 | 51,165,268 | 50,230,598 | 4,740,008 |
|  | % | 97.998 | 98.170 | 97.824 | 0.665 |
|  | % of bacteria classified^♣^ | 57.716 | 59.610 | 55.821 | 5.459 |
|  | % of archaea classified^♣^ | 3.496 | 2.658 | 4.334 | 0.560 |
| rRNAs | No. of seqs | 44,583,023 | 44,302,196 | 44,863,850 | 5,433,677 |
|  | % | 86.178 | 85.002 | 87.372 | 3.510 |
| non-rRNA genes | No. of seqs | 6,114,910 | 6,863,072 | 5,366,748 | 1,871,120 |
|  | % | 11.820 | 13.168 | 10.452 | 4.009 |
| Deduced amino acid sequences | No. of seqs | 294,295 | 377,587 | 211,003 | 82,075 |
|  | % | 0.608 | 0.738 | 0.479 | 0.181 |
|  | % of non-rRNAs | 4.813 | 5.502 | 3.932 | 0.949 |
| Annotated  KEGG orthologs | No. of seqs | 117,951 | 138,009 | 97,893 | 33,018 |
|  | % | 0.242 | 0.263 | 0.220 | 0.072 |
|  | % of non-rRNAs | 1.929 | 2.011 | 1.824 | 0.124 |

*, Quality-filtered (>Q25) 16S amplicon sequences were used in our previous study [1].

#, percentage of total RNA sequences.

♣, percentage of host-filtered sequences.

**Table S4.** Alpha diversity measurements of bacterial and archaeal genera in microbial inocula.

| **Bacterial alpha-diversity** | | | | |
| --- | --- | --- | --- | --- |
| Inoculum | Bacteria-enriched inoculum | Protozoa-enriched inoculum | SEM | *P*-value |
| Observed genera | 762 | 603 | 64.920 | 0.354 |
| Chao1 estimates | 971 | 742 | 87.330 | 0.346 |
| Evenness | 0.285 | 0.285 | 0.013 | 0.992 |
| Shannon's index | 2.725 | 2.618 | 0.115 | 0.713 |
| Simpson's index | 0.708 | 0.699 | 0.047 | 0.892 |
| **Archaeal alpha-diversity** | | | | |
| Inoculum | Bacteria-enriched inoculum | Protozoa-enriched inoculum | SEM | *P*-value |
| Observed genera | 14 | 11 | 1.181 | 0.145 |
| Chao1 estimates | 15 | 14 | 1.887 | 0.771 |
| Evenness | 0.255 | 0.160 | 0.047 | 0.011 |
| Shannon's index | 0.972 | 0.553 | 0.188 | 0.017 |
| Simpson's index | 0.356 | 0.186 | 0.084 | 0.062 |

**References**

1. Park T, Cersosimo LM, Li W, Radloff W, Zanton GI: **Pre-weaning ruminal administration of differentially-enriched, rumen-derived inocula shaped rumen bacterial communities and co-occurrence networks of post-weaned dairy calves.** *Front Microbiol* 2021, **12:**311.
